# Harm reduction in name, but not substance: a comparative analysis of current Canadian provincial and territorial policy frameworks

**DOI:** 10.1186/s12954-017-0177-7

**Published:** 2017-07-26

**Authors:** Elaine Hyshka, Jalene Anderson-Baron, Kamagaju Karekezi, Lynne Belle-Isle, Richard Elliott, Bernie Pauly, Carol Strike, Mark Asbridge, Colleen Dell, Keely McBride, Andrew Hathaway, T. Cameron Wild

**Affiliations:** 1 School of Public Health, University of Alberta, 3-300 Edmonton Clinic Health Academy, 11405 87 Avenue NW, Edmonton, Alberta T6G 1C9 Canada; 2 Inner City Health and Wellness Program, B818 Women’s Centre, Royal Alexandra Hospital, 10240 Kingsway Avenue, Edmonton, Alberta T5H 3V9 Canada; 3 Canadian AIDS Society, 190 O’Connor St., Suite 100, Ottawa, Ontario K2P 2R3 Canada; 4 Canadian HIV/AIDS Legal Network, 1240 Bay St., Suite 600, Toronto, Ontario M5R 2A7 Canada; 5 School of Nursing and Centre for Addictions Research of BC, University of Victoria, Box 1700 STN CSC, Victoria, British Columbia V8W 2Y2 Canada; 6 Dalla Lana School of Public Health, University of Toronto, 155 College Street, Toronto, Ontario M5T 3M7 Canada; 7 Department of Community Health and Epidemiology, Centre for Clinical Research, Dalhousie University, Room 407, 5790 University Ave, Halifax, Nova Scotia B3H 1V7 Canada; 8 Department of Sociology, University of Saskatchewan, Room 1109–9 Campus Drive, Saskatoon, Saskatchewan S7N 5B5 Canada; 9 Addiction and Mental Health Branch, Health Service Delivery Division, Alberta Health Services, P.O. Box 1360, Station Main, Edmonton, Alberta T5J 2N3 Canada; 10 Department of Sociology, University of Guelph, 50 Stone Rd E, Guelph, Ontario N1G 2W1 Canada

**Keywords:** Harm reduction, Policy, Content analysis, Canada

## Abstract

**Background:**

In Canada, funding, administration, and delivery of health services—including those targeting people who use drugs—are primarily the responsibility of the provinces and territories. Access to harm reduction services varies across jurisdictions, possibly reflecting differences in provincial and territorial policy commitments. We examined the quality of current provincial and territorial harm reduction policies in Canada, relative to how well official documents reflect internationally recognized principles and attributes of a harm reduction approach.

**Methods:**

We employed an iterative search and screening process to generate a corpus of 54 provincial and territorial harm reduction policy documents that were current to the end of 2015. Documents were content-analyzed using a deductive coding framework comprised of 17 indicators that assessed the quality of policies relative to how well they described key population and program aspects of a harm reduction approach.

**Results:**

Only two jurisdictions had current provincial-level, stand-alone harm reduction policies; all other documents were focused on either substance use, addiction and/or mental health, or sexually transmitted and/or blood-borne infections. Policies rarely named specific harm reduction interventions and more frequently referred to generic harm reduction programs or services. Only one document met all 17 indicators. Very few documents acknowledged that stigma and discrimination are issues faced by people who use drugs, that not all substance use is problematic, or that people who use drugs are legitimate participants in policymaking. A minority of documents recognized that abstaining from substance use is not required to receive services. Just over a quarter addressed the risk of drug overdose, and even fewer acknowledged the need to apply harm reduction approaches to an array of drugs and modes of use.

**Conclusions:**

Current provincial and territorial policies offer few robust characterizations of harm reduction or go beyond rhetorical or generic support for the approach. By endorsing harm reduction in name, but not in substance, provincial and territorial policies may communicate to diverse stakeholders a general lack of support for key aspects of the approach, potentially challenging efforts to expand harm reduction services.

**Electronic supplementary material:**

The online version of this article (doi:10.1186/s12954-017-0177-7) contains supplementary material, which is available to authorized users.

## Background

Canadian support for harm reduction has waxed and waned with prevailing political winds. However, the election of a new federal government in 2015 and a dramatic rise in overdose deaths in multiple provinces and territories has made implementation of new harm reduction policies and related services likely. In an effort to inform future policy development, we examine the current state of provincial and territorial policy frameworks, with the aim of systematically describing the quality of policies, relative to their communicative functions. We conclude with recommendations for how future policies could better reflect internationally recognized understandings of harm reduction.

### Brief history of harm reduction in Canada

During the late 1980s, Canada, alongside Australia and a number of Western European countries, became an early pioneer of contemporary harm reduction approaches in response to rising rates of HIV infection among people who inject drugs [1]. Peer-driven and informal syringe distribution emerged in Montreal, Toronto, and Vancouver in 1988, and in 1989, the federal Department of Health partnered with five provinces to implement eight official programs in major Canadian cities [2–4]. When federal funding ended two years later, many of these programs continued with support from the provinces [2], and today, most of Canada’s 13 provinces and territories have syringe distribution programs [5, 6]. Federal support for harm reduction continued until the mid 2000s and included the recognition of harm reduction as a key pillar of federal drug policy [7], attempts to decriminalize cannabis possession [8], and the granting of two temporary legal exemptions allowing for the opening of Insite, North America’s first supervised injection facility, without risk of criminal prosecution under federal drug laws, [9] and the implementation of Canada’s first heroin-assisted treatment clinical trial [10].

This progress stalled in 2007 when a recently elected Conservative government replaced Canada’s Drug Strategy with a new National Anti-drug Strategy, transferred lead responsibility for drug policy from the Department of Health to the Department of Justice, and officially excised harm reduction from federal policy [11]. The new federal health minister made it clear the government was highly unlikely to renew Insite’s legal exemption [12], and publicly declared the facility “an abomination” (during the 2008 International AIDS Conference) [13, 14]. Insite’s operators and clients challenged the minister’s failure to renew their exemption in court, arguing that their rights to life, liberty, and security of the person under the *Canadian Charter of Rights and Freedoms* had been infringed. This legal action culminated in a 2011 Supreme Court of Canada ruling ordering the Minister of Health to renew Insite’s exemption [15]. In response, the federal government enacted a new, highly restrictive legislative framework that created an onerous process for obtaining an exemption for a supervised injection facility to operate without risk of prosecution [16]. Beyond opposition to supervised injection, the government also legislated new mandatory minimum sentences for certain drug offenses [17], rejected growing calls to implement syringe distribution programs in federal correctional facilities [18], and introduced a number of roadblocks to legal access to prescription heroin under Health Canada’s Special Access Programme [19].

Political hostility to harm reduction at the federal level meant that during the Conservative government’s 10 years in power, stewardship of Canadian harm reduction policy fell almost entirely under the purview of the provinces and territories. In Canada, the federal government and the provinces and territories have shared responsibility over health. Each province and territory has legislation governing its health system, which provides universal, publicly funded access to hospital and physician services to citizens [20]. Funding, administration, and delivery of health services—including those targeting people who use drugs—are thus primarily the responsibility of the provinces and territories. Yet, the federal government also plays a role by making monetary transfers for provincial and territorial health expenditures; many of which are contingent on broad service criteria. The federal Department of Health also sets national policy direction (including allocating resources) for epidemiological surveillance and other public health programs, health protection, and food and drug regulation [20].

Shared jurisdiction over health and primary responsibility for the delivery of services meant that provinces and territories could continue to articulate formal policy and funding commitments to harm reduction despite federal opposition to the approach. Yet, access to Canadian harm reduction services remains highly variable across jurisdictions, perhaps reflecting inconsistent provincial and territorial political support for the approach. Until the end of 2016, supervised injection facilities were only available in one city, Vancouver, British Columbia (several more facilities have since opened in other parts of British Columbia, and three sites in Montreal are operational as of this writing [21]); take-home naloxone programs had only recently expanded beyond Alberta, British Columbia, and Ontario [22]; and in some parts of the country, syringe distribution programs remain unavailable [5, 6]. This variation in service availability suggests that to date, some provincial and territorial policy frameworks have not been sufficiently robust to support consistent uptake and scale-up of harm reduction across the country.

The end of 2015 introduced a new era for harm reduction in Canada, including the prospect of significant expansion of services for people who use drugs. A number of factors converged to open this current policy window [23]. The election of a new Liberal majority government in October 2015 ended a decade of federal antagonism towards harm reduction, and signaled the willingness of the federal government to support provincial and territorial efforts to improve access to these services. During the election campaign, the Liberal Party of Canada explicitly endorsed supervised injection facilities [24] and prison syringe distribution programs, [25] and promised to legalize and regulate cannabis [26]. By the end of 2016, the Liberal government had taken action to ease restrictions on heroin-assisted treatment [27] and supervised injection facilities, [28] put in place by the previous government, and returned harm reduction to federal policy by declaring a new *Canadian Drugs and Substances Strategy*, with lead responsibility for the strategy returned to the Department of Health [29].

Beyond a shift in political winds, an ongoing overdose epidemic has brought dramatic increases in the number of opioid-related deaths to several parts of the country [30, 31]. The western provinces have been significantly impacted [31], as evidenced by a 79% year-over-year increase in the number of overdose deaths recorded in British Columbia in 2016, resulting in a rate of 20.4 deaths per 100,000 population [32]. In Alberta, complete provincial 2016 overdose statistics have not been released as of this writing. However, partial reports indicate that 363 Albertans died of an apparent overdose related to fentanyl in 2016, and 196 died of an apparent overdose related to an opioid other than fentanyl, resulting in death rates of 8.6 and 4.6 per 100,000 population, respectively [33]. Such statistics are prompting provinces and territories to expand access to take-home naloxone [22, 34] and consider implementing additional harm reduction programs as one strategy for preventing or mitigating similar crises within their own jurisdictions [35, 36]. Renewed interest in harm reduction federally and within the provinces and territories also reflects the influence of researchers, healthcare providers, advocacy groups, and organizations of people who use drugs who are pressuring governments to significantly strengthen existing harm reduction approaches [37–39]. The current policy window therefore portends the development of new harm reduction policies and programs for addressing illegal drug use in Canada. As Canada embarks on this new era, there is a need to: take stock of provincial and territorial harm reduction policy frameworks within which future policies and programs may be developed and implemented; assess the quality of current policy; and outline directions for future policy development.

### Rationale

The present study is a component of the Canadian Harm Reduction Policy Project (CHARPP) and attends to this analytic task. CHARPP is a mixed-method, multiple case study drawing on four data sources (policy documents, key informant interviews, media texts, and a national public opinion survey) to analyze how policies governing harm reduction services are positioned within and across the Canadian provinces and territories. Rather than a review of frontline services, CHARPP is examining Canadian harm reduction governance and policymaking, including laws, rules, policies, and administrative practices that constrain, prescribe, and enable the provision of public goods and services [40]. In decentralized federations such as Canada, where most health system decision-making is devolved to subnational regional governments, policy frameworks within these jurisdictions are particularly central to understanding availability of health services therein [20]. Thus, one of CHARPP’s initial research undertakings was a systematic search and comparative analysis of formal policy used for the planning and delivery of harm reduction services across Canada’s provinces and territories.

This paper reports the results of a comprehensive search and comparative analysis of Canadian harm reduction policy documents. In a previous analysis, we used Lynn et al.’s [40] reduced form logic of governance to describe Canadian harm reduction policy texts in relation to the strength of their instrumental functions [41]. This included assessing the extent to which policies reference legislation, are endorsed by elected officials, indicate funding commitments and timelines, and assign responsibilities to specific actors. We found that historically, across provincial and territorial policy frameworks, few policies articulate specific managerial or structural components to guide comprehensive, accountable, and transparent governance of harm reduction services in Canada [41]. Although useful for evaluating features of governance in a specified policy domain, a weakness of Lynn et al.’s [40] logic of governance is that it does not refer to communicative functions of policy. This is problematic because formal policy documents are also government communication tools which convey underlying normative assumptions and conceptual logics of policy problems [42, 43]. In deliberately presenting specific knowledge, values, and beliefs, these texts reinforce to an audience of diverse government and societal actors preferred understandings of illegal drug use, harm reduction, and other policy responses [42]. Accordingly, the present analysis complements our previous work by describing how provincial and territorial policy documents conceptualize harm reduction as an approach to illegal drug use, and the extent to which these understanding reflect internationally recognized attributes and principles of harm reduction.

## Methods

### Document retrieval and screening

Our systematic search strategy, comprehensive inclusion and exclusion parameters, and screening methods have been described in detail elsewhere [41] and are thus reviewed only briefly here. We employed an iterative search and screening process to generate a corpus of policy texts for the present analysis. Boolean searches were conducted by entering 13 separate search vocabularies into an Internet search engine (Google) to retrieve publicly available policy documents related to harm reduction from each province (*n* = 10) and territory (*n* = 3). We limited our search to documents published by provincial and territorial governments or their delegated health authorities between 2000 and the end of 2015. Temporal parameters were mainly determined on pragmatic grounds, i.e. to make the review a manageable process and to ensure documents reflected policies produced across government transitions at the federal and provincial levels. In each province, regional health authority restructuring since 2000 combined with a general lack of availability of archival documents on the Internet made a national in scope search of documents authored prior to 2000 largely unfeasible.

Our initial search yielded 522 documents, which were screened for relevance. In line with Ritter and Berends [44], we defined relevant documents as harm reduction policy texts that (1) were issued by and representing a provincial or territorial government or (2) issued by and representing a regional, provincial, or territorial delegated health authority; (3) mandated future action; (4) addressed harm reduction services and interventions, defined as one or more of the following: syringe distribution, naloxone, supervised injection/consumption, safer inhalation kits, low-threshold opioid agonist (i.e., methadone) treatment, buprenorphine/naloxone (Suboxone), and drug checking services; or (5) was produced as either a stand-alone harm reduction policy or as part of a strategy document guiding services for substance use, addiction, mental health, and/or prevention of blood-borne or sexually transmitted infections. We excluded documents that described services at the municipal level, in prisons (which have been described elsewhere [45, 46]), and on First Nation reserves (where health services are the responsibility of the federal government). Additionally, given our focus on provincial and territorial *policy* frameworks, and not on aspects of harm reduction *practice*, we excluded government or health authority-authored documents exclusively focused on best practice guidelines for frontline service providers.

A total of 81 documents met our inclusion criteria after our initial search process. We then undertook purposive searches for progress updates or status reports for all 81 included policy documents. These additional searches yielded another 20 texts for analysis. We circulated the list of included documents to our CHARPP national reference committee—which comprises 76 policymakers, service providers, and researchers with an interest in harm reduction policy from across the provinces and territories. Feedback from this group verified the accuracy of our initial results and generated one additional text for inclusion in our corpus, resulting in 102 documents. Finally, prior to analysis, we classified each policy document as either “current” or “historical.” Documents were classified as current if (1) the policy was in effect in 2015; (2) the document was the most recent version retrieved for the case and had not been replaced by a newer document with the same focus; and/or (3) the document had no stated end date. In total, we classified 54 documents as “current.” The remaining documents were classified as historical and are not included in the present analysis.

### Document analysis

We developed the CHARPP framework to guide deductive coding of documents and facilitate systematic comparative analysis of policies included in our review.

#### CHARPP framework

Policy quality can be measured according to multiple dimensions. The CHARPP coding framework is a set of indicators designed to assess the quality of harm reduction policies based on the strength of their communicative functions. Formal policies that score highly on CHARPP indicators are of high quality because they conceptualize and describe a harm reduction approach in close accordance with its internationally recognized attributes and principles [47, 48]. Conversely, poor-quality harm reduction policies score low on CHARPP indicators because they refer to the approach only sparingly and/or do not elucidate its key attributes and principles. To build the CHARPP framework, we generated a preliminary list of indicators based on key harm reduction principles and attributes outlined by Harm Reduction International’s *What is harm reduction?* position statement [47]. Harm Reduction International is a leading non-governmental organization working to promote and expand support for harm reduction globally. Biennially, they publish the “Global State of Harm Reduction Report,” which collates information on harm reduction policies and programs across global regions. The World Health Organization’s “Consolidated guidelines on HIV prevention, diagnosis, treatment, and care for key populations” were also referenced [48]. Once our preliminary indicator list was generated, CHARPP’s investigator group (including 14 harm reduction experts with academic, government, or non-profit backgrounds) revised and refined it to ensure it reflected what would be considered quality indicators of harm reduction policy in the Canadian context. Our final coding framework is outlined in Table 1 and includes 17 indicators grouped according to whether policies adequately specified *population* characteristics or *program* features. We constructed the CHARPP indicators using statements that facilitated dichotomous (yes/no) coding, enabling direct comparisons of documents and facilitating ranking of cases.

**Table 1:** CHARPP coding framework for assessing quality of harm reduction policies

Population quality indicators
Includes 9 population indicators based on the premise that high-quality harm reduction policies characterize service populations accurately when they: 1. Recognize that stigma and/or discrimination are issues faced by people who use illicit drugs 2. Affirm that people who use drugs need to be involved in policy development or implementation 3. Acknowledge that not all substance use is problematic 4. Recognize that harm reduction has benefits for both people who use drugs and the broader community 5. Acknowledge that a harm reduction approach can be applied to the general population 6. Affirm that women are a key population 7. Affirm that youth are a key population 8. Affirm that indigenous people are a key population 9. Affirm that one or more groups of LGBTQI (lesbian, gay, bisexual, trans, queer and questioning, and intersex) people are a key population
Program quality indicators
Includes 8 program indicators based on the premise that high-quality harm reduction policies should: 10. Acknowledge the need for evidence-informed policies and/or programs 11. Recognize the importance of preventing drug-related harm (rather than just preventing drug use, or blood-borne, or sexually transmitted infections) 12. Discuss low-threshold [72] approaches to service provision 13. Specifically address overdose 14. Recognize that reducing or abstaining from substance use is not required 15. Consider harm reduction approaches for a variety of drugs and modes of use 16. Discuss harm reduction’s human rights (e.g., dignity, autonomy) dimensions 17. Consider the social determinants (including income, housing, and education) that influence drug-related harm

In addition to these 17 indicators, for each document, we noted publication date, scope (provincial/territorial or delegated health authority), document focus (harm reduction, addiction/mental health/substance use, or sexually transmitted and blood-borne infection prevention), and number of mentions of specific harm reduction interventions.

#### Analysis

Each document was reviewed for the presence (1 = yes, criteria met) or absence (0 = no, criteria not met) of each quality indicator. Dichotomous scores for each indicator were justified with an accompanying written rationale. Scores and rationales were then compiled into a standardized *policy report card* for each provincial or territorial case to facilitate comparisons of harm reduction policy across jurisdictions. Each report card was then assessed amongst the authors doing the rating (JA-B, KK, LBI) for agreement. Disagreements were resolved through discussion with EH, as required. Once finalized, indicator scores from all 13 policy report cards were compiled into a single dataset to facilitate across-case comparisons (Additional file 1).

To characterize each case’s policy documents, we calculated the total number of document pages per jurisdiction and as a proportion of the total number of pages analyzed overall. We also computed the total number of times, and the rate per document, that specific harm reduction interventions were mentioned for each case. To facilitate comparisons across cases relative to the quality of their harm reduction policy, we first computed a percentage score across the nine population indicators for each case as the sum of all “criterion met” (=1) scores relative to the total possible score (9 × no. of documents in the case). We repeated this procedure for the eight program indicators. Finally, we computed a total percentage score across all 17 (nine population and eight program) CHARPP indicators to facilitate an overall ranking of the cases. The closer a given case’s percentage score was to 100%, the greater the number of policy documents in the case that exemplify high-quality harm reduction policy. Finally, to synthesize our case-level findings and evaluate the overall quality of provincial and territorial policy across Canada, we computed percentage scores for each indicator across all 54 current provincial and territorial documents.

## Results

### Document characteristics

As shown in Table 2, we retrieved a total of 54 current policy documents for the 13 provinces and territories, with a mean of four documents per case and a range of zero (Yukon Territory) to 11 (Quebec). Two cases, Quebec and British Columbia, accounted for 39% of all current policy documents.

**Table 2:** Harm reduction policy documents by case (*N* = 54)

Case	Total no. of current documents (% of Canadian total)	Total no. of pages (% of Canadian total)
British Columbia	10 (19)	447 (15)
Alberta	4 (7)	246 (9)
Saskatchewan	3 (6)	447 (15)
Manitoba	7 (13)	142 (5)
Ontario	7 (13)	336 (12)
Quebec	11 (20)	544 (19)
New Brunswick	1 (2)	24 (1)
Nova Scotia	4 (7)	352 (12)
Prince Edward Island	1 (2)	26 (1)
Newfoundland and Labrador	2 (4)	164 (6)
Yukon	0 (0)	0 (0)
Northwest Territories	2 (4)	72 (2)
Nunavut	2 (4)	91 (3)
Canada	54 (100)	2891 (100)

Document publication years ranged from 2001 to 2015, with 80% of all documents published between 2006 and 2015. Only two jurisdictions, British Columbia and Alberta, had current stand-alone, provincial-level harm reduction policies (Manitoba had two regional-level stand-alone harm reduction policies, but no provincial one). All other documents were focused on either substance use, addiction and/or mental health (*n* = 35), or sexually transmitted and/or blood-borne infections (*n* = 15).

### Harm reduction interventions

Table 3 outlines the frequency and rate per document of mentions of various harm reduction interventions across the provincial and territorial cases. Generic, non-specific references to “harm reduction” appeared an average of 13.9 times per document. The most common specific interventions mentioned were syringe distribution programs (4.0 times/document) and supervised injection or consumption (1.5 times/document). Two cases (New Brunswick and the Northwest Territories) made no mention of the term harm reduction or any specific harm reduction interventions in their current policy documents. Ontario, Canada’s largest province with a population of almost 14 million, discussed harm reduction sparingly and only mentioned one specific harm reduction intervention—syringe distribution programs—once in current policy. Saskatchewan was the province or territory that most frequently mentioned “syringe distribution” (36.3 times/document) and supervised injection or consumption (12.7 times/document) in its formal policy documents.

**Table 3:** Number (and rate) of specific harm reduction interventions identified in provincial and territorial policy documents

Case (no. of documents within cases)	Harm reduction (unspecified)	Needle/syringe distribution	Naloxone	Supervised injection or consumption	Low-threshold opioid agonist treatment	Buprenorphine/naloxone (Suboxone)	Drug checking	Safer inhalation kits
British Columbia (10)	208 (20.8)	31 (3.1)	6 (0.6)	22 (2.2)	0 (0.0)	1 (0.1)	0 (0.0)	3 (0.3)
Alberta (4)	78 (19.5)	9 (2.3)	0 (0.0)	2 (0.5)	0 (0.0)	0 (0.0)	0 (0.0)	1 (0.3)
Saskatchewan (3)	253 (84.3)	109 (36.3)	0 (0.0)	38 (12.7)	0 (0.0)	0 (0.0)	0 (0.0)	1 (0.3)
Manitoba (7)	80 (11.4)	13 (1.9)	1 (0.1)	2 (0.3)	0 (0.0)	0 (0.0)	1 (0.1)	2 (0.3)
Ontario (7)	9 (1.3)	1 (0.1)	0 (0.0)	0 (0.0)	0 (0.0)	0 (0.0)	0 (0.0)	0 (0.0)
Quebec (11)	42 (3.8)	11 (1.0)	0 (0.0)	17 (1.5)	5 (0.5)	0 (0.0)	1 (0.1)	0 (0.0)
New Brunswick (1)	0 (0.0)	0 (0.0)	0 (0.0)	0 (0.0)	0 (0.0)	0 (0.0)	0 (0.0)	0 (0.0)
Nova Scotia (4)	63 (15.8)	38 (9.5)	2 (0.5)	0 (0.0)	1 (0.3)	27 (6.8)	0 (0.0)	0 (0.0)
Prince Edward Island (1)	6 (6.0)	0 (0.0)	0 (0.0)	0 (0.0)	0 (0.0)	0 (0.0)	0 (0.0)	0 (0.0)
Newfoundland and Labrador (2)	5 (2.5)	2 (1.0)	0 (0.0)	0 (0.0)	0 (0.0)	0 (0.0)	0 (0.0)	0 (0.0)
Yukon (0)	n/a	n/a	n/a	n/a	n/a	n/a	n/a	n/a
Northwest Territories (2)	0 (0.0)	0 (0.0)	0 (0.0)	0 (0.0)	0 (0.0)	0 (0.0)	0 (0.0)	0 (0.0)
Nunavut (2)	9 (4.5)	0 (0.0)	0 (0.0)	0 (0.0)	0 (0.0)	0 (0.0)	0 (0.0)	0 (0.0)
Canada (54)	753 (13.9)	214 (4.0)	10 (0.2)	81 (1.5)	6 (0.1)	28 (0.5)	2 (0.04)	7 (0.1)

*n/a* not applicable

### Harm reduction policy quality: population characteristics

**Fig. 1:**
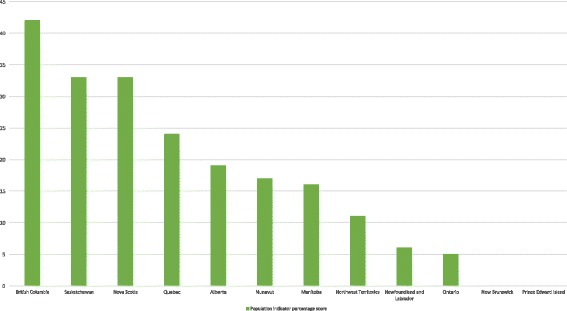
Percentage scores on CHARPP population indicators by province or territory. All current harm reduction policy documents in a given jurisdiction were coded on 9 population quality indicators (1 = indicator met; 0 = indicator not met). We added together all population indicator scores of “1” for each case and divided by the potential total (9 × no. of documents in the case), resulting in a percentage score on population quality that could easily be compared between cases. The higher the case’s overall percentage score (out of a maximum of 100), the greater the number of policy documents in the case that exemplify high-quality harm reduction policy. Note that the Yukon Territory had no current harm reduction policy documents and is thus excluded from this figure. New Brunswick and Prince Edward Island had current harm reduction policy documents; however, none met any of our population indicators

Figure 1 shows provincial and territorial scores on our set of 9 population quality indicators. British Columbia (42%), Saskatchewan (33%), and Nova Scotia (33%) performed best overall on the population indicators. None of the current policy documents in two Atlantic provinces—New Brunswick and Prince Edward Island—met any population quality indicators. Across all included documents, the average on the eight population indicators was 22%.

Across cases (Additional file 1), one out of 54 harm reduction policy documents met all quality indicators related to populations: British Columbia’s *BC Harm Reduction Strategies and Services Policy and Guidelines 2014* [49]. Many documents (*n* = 20) had no positive scores on any of the population indicators. Recognizing that stigma and discrimination are issues faced by people who use illegal drugs (indicator 1) was the population indicator most frequently mentioned across cases (39% of documents). With regard to other indicators related to populations, 31% of documents affirmed that people who use substances need to be involved in policy development or implementation (indicator 2) and 22% of documents acknowledged that not all substance use is problematic (indicator 3). Least frequently mentioned were the population indicators relating to harm reduction for specific key populations: Indigenous people (indicator 9) were mentioned in 17% of documents, women (indicator 6) were mentioned in 11% of documents, and LGBTQI people (indicator 10) were mentioned in 9% of documents.

### Harm reduction policy quality: program features

**Fig. 2:**
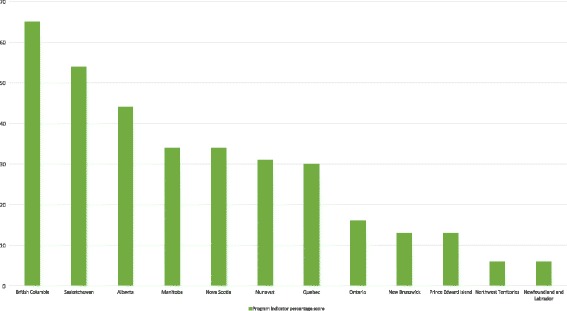
Percentage scores on CHARPP program indicators by province or territory. All current harm reduction policy documents in a given jurisdiction were coded on 8 program quality indicators (1 = indicator met; 0 = indicator not met). We added together all program indicator scores of “1” for each case and divided by the potential total (8 × no. of documents in the case), resulting in a percentage score on program quality that could easily be compared between cases. The higher the case’s overall percentage score (out of a maximum of 100), the greater the number of policy documents in the case that exemplify high-quality harm reduction policy. Note that the Yukon Territory had no current harm reduction policy documents and is thus excluded from this figure

Almost all cases scored higher on program indicators than they did on population indicators. British Columbia (65%), Saskatchewan (54%), and Alberta (44%) performed best overall on this front (Fig. 2). Two Atlantic provinces (New Brunswick and Prince Edward Island) each scored 13%, with a third Atlantic province (Newfoundland and Labrador) and the Northwest Territories ranking lowest overall with 6%. The average score on program quality indicators across all included documents was 35%.

Five policy documents, including three from British Columbia, one from Saskatchewan, and one from Manitoba, met all program quality indicators, and eight documents did not meet any program quality indicators (Additional file 1). Acknowledging the need for evidence-informed policies and/or programming (indicator 10) was by far the most frequently endorsed amongst all eight program quality indicators: 70% of documents had positive scores. Less than 40% of documents discussed the need for low-threshold or low-barrier approaches to service provision (indicator 12, 37%) or acknowledged the need to reduce substance-related harm (not just substance use or sexually transmitted or blood-borne infections) (indicator 11, 39%). Finally, less than 30% discussed the need to address the risk of overdose (indicator 13, 28%) or considered harm reduction approaches for a variety of types of drugs or modes of use (indicator 15, 19%).

### Harm reduction policy quality: overall performance

**Fig. 3:**
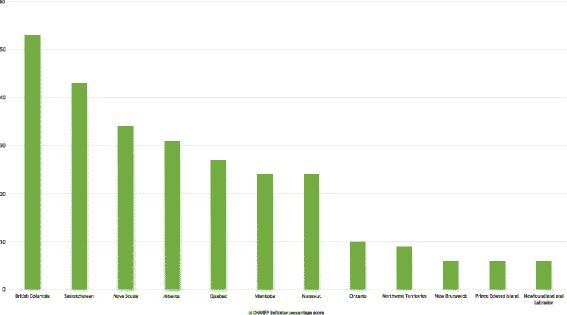
Percentage scores on all 17 CHARPP population and program indicators by province or territory. All current harm reduction policy documents in a given jurisdiction were coded on 9 population indicators and 8 program quality indicators (1 = indicator met; 0 = indicator not met). We added together all 17 indicator scores of “1” for each case and divided by the potential total (17 × no. of documents in the case), resulting in a cumulative percentage score on quality that could easily be compared between cases. The higher the case’s overall percentage score (out of a maximum of 100), the greater the number of policy documents in the case that exemplify high-quality harm reduction policy. Note that the Yukon Territory had no current harm reduction policy documents and is thus excluded from this figure

Across all 17 CHARPP indicators, British Columbia, Saskatchewan, and Nova Scotia had the highest quality formal harm reduction policies, while the Northwest Territories and three Atlantic provinces (Prince Edward Island, New Brunswick, and Newfoundland and Labrador) performed poorest overall (Fig. 3). The average score across all included documents on the 17 CHARPP indicators was 29%.

### Formal definitions of harm reduction

The concept of harm reduction was rarely formally defined in current policies. New Brunswick, Prince Edward Island, Newfoundland and Labrador, and Northwest Territories offered no formal harm reduction definitions within their policy frameworks. Only British Columbia and Alberta provided formal definitions of harm reduction in the majority of their current policy documents. We found that harm reduction definitions within each of these provinces’ policy documents tended to overlap and were fairly consistent. However, both provinces defined the concept somewhat differently. Table 4 demonstrates how formal definitions of harm reduction (when available) vary across provinces and territories. Many of the definitions provided do incorporate some key principles and attributes of harm reduction.

**Table 4:** Examples of formal harm reduction definitions found within provincial and territorial policy documents

British Columbia (8/10 documents formally define harm reduction)
“Harm reduction refers to policies, programs, and practices that seek to reduce the adverse health, social and economic consequences of the use of legal and illegal psychoactive substances. Harm reduction is pragmatic and focuses on keeping people safe and minimizing death, disease and injury associated with higher risk behaviour, while recognizing that the behaviour may continue despite the risks. It seeks to lessen the harms associated with substance use while recognizing that many individuals may not be ready or in a position to cease use. Harm reduction does not require, nor does it exclude, abstinence from drug use as an ultimate goal. Harm reduction is an essential part of a comprehensive public health response to problematic substance use that complements prevention, treatment and enforcement. A harm reduction philosophy should inform strategies directed at the whole population, as well as specific programs aimed at sub-populations of vulnerable people. Harm reduction acknowledges the ethical imperative of helping to keep people as safe and healthy as possible, while upholding human rights, respecting individual autonomy and supporting informed decision making in the context of active substance use.”—*BC Harm Reduction Strategies and Services Policy and Guidelines* 2014, p. 2
Alberta (3/4 documents formally define harm reduction)
“Harm reduction recognizes there will always be a portion of the population who will engage in higher risk behaviours, such as the use of unprescribed injection drugs and/or have unprotected sex with more than one sex partner. Harm reduction focuses on reducing or minimizing the harms associated with higher-risk behaviours. Harm reduction helps protect individuals from the most harmful health consequences of addiction behaviours for themselves, their families / partners and their communities, while facilitating referrals to treatment and rehabilitation services.”—*Alberta Sexually Transmitted Infections and Blood Borne Pathogens Strategy and Action Plan*, p. 19
Saskatchewan (1/3 documents formally define harm reduction)
“Harm reduction: (1) is an approach or strategy that aims to reduce the negative consequences of drug use, rather than to eliminate drug use; (2) can involve programs or policies that are designed to reduce drug-related harm without requiring abstinence or cessation of drug use; (3) promotes incremental improvements in the behaviours of injection drug users that are practical, achievable and ultimately lead to benefits for both users and communities.”—*Building Partnerships for Health: A Strategic Planning Framework for Injection Drug Use in Saskatoon*, p. 105
Manitoba (1/3 documents formally define harm reduction)
“Harm reduction strategies engage people who are at risk of contracting HIV or hepatitis C, focusing on where they are in their lives. It is a pragmatic approach that recognizes the limitations of abstinence-based approaches for populations with well-entrenched high-risk behaviour patterns. Harm reduction approaches focus on decreasing the negative consequences of high-risk behaviours to individuals, communities and society. Rather than necessarily attempting to have people cease engaging in behaviours that are associated with the spread of HIV (such as sharing injection drug equipment and unprotected sexual contact), it seeks to reduce the potential harm of such activities. These strategies may result in some people abstaining from risk behaviours; however, abstinence is not the primary objective of harm reduction. The focus is on assisting people to change their risk behaviours through education, peer support and opportunity building. Harm reduction strategies can include confidential condom provision; needle distribution and exchange; safe disposal sites for used injection equipment; safe injection sites; a policing focus on drug dealers; and pharmacy, health centre, nursing station and community involvement in needle exchange and sales. They can also include media campaigns focusing on the continuing prevalence and risk of HIV, sexually transmitted disease and hepatitis C infection, prevention and harm reduction activities and the benefits of early testing and treatment in reducing transmission to others and improving quality of life for persons with HIV/AIDS. Harm reduction approaches must recognize and be sensitive to Aboriginal cultural diversity, the traumatic effects of attempted assimilation and the unique aspects of post-colonial cultural revitalization.”—*As Long as the Waters Flow: An Aboriginal Strategy on HIV/AIDS. A Component of Manitoba’s Provincial AIDS Strategy*, p. 24
Ontario (1/7 documents formally define harm reduction)
“… any program or policy designed to help reduce substance-related harm without requiring the cessation of substance use”—*Ontario Public Health Standards*, p. 32
Quebec (2/11 documents formally define harm reduction)
“It is impossible to eliminate the use of illegal drugs or the problematic use of alcohol as much as it is to think we can eliminate heart disease or cancer. It is possible, however, to limit or reduce the health and well-being problems as well as the harms that result from the inappropriate use of psychoactive substances. This means that in addition to health promotion and prevention measures, services must be offered to people who use drugs that, without aiming for abstinence or non-use, aim to reduce the harms associated with inappropriate use.” [translation]—*Pour une Approche Pragmatique de Prévention en Toxicomanie: Orientations, Axes d’intervention*, *Actions*, p. 25
Nova Scotia (2/4 documents formally define harm reduction)
“Policies, programs, and practices that aim to reduce the negative health, social, and economic consequences (e.g., HIV, hepatitis B and C, overdoses) that may ensue from the use of legal and illegal psychoactive drugs without necessarily reducing or stopping drug use. Its cornerstones are public health, human rights, and social justice. It benefits people who use drugs, families, and communities. It ensures that people who use psychoactive substances are treated with respect and without stigma and that substance-related problems and issues are addressed systemically.”—*Review of Nova Scotia’s Strategy on HIV/AIDS: Looking Back & Moving Forward*, p. 59
Nunavut (1/2 documents formally define harm reduction)
Using illicit drug use as an example, characteristics or principles of harm reduction are as follows: 1) pragmatism; 2) focus on harms; 3) balancing costs and benefits; 4) priority of immediate goals. Under these four principles there is extensive information included for each. In general, pragmatism, respect and dignity for the person using drugs, focusing on harms, and setting incremental and realistic immediate goals are key aspects of harm reduction that are emphasized. Importantly it is noted that “containment and amelioration of drug related harms may be a more pragmatic or feasible option than efforts to eliminate drug use entirely.” The authors also note this model supports a hierarchy of goals, and abstinence as a possible goal within this hierarchy “Most harm-reduction programs have a hierarchy of goals, with the immediate focus on proactively engaging. Achieving the most immediate and realistic goals is usually viewed as first steps toward risk-free use, or, if appropriate, abstinence for individuals, target groups, and communities to address their most pressing needs.”—*Nunavut Addiction and Mental Health Strategy*, p. 59

New Brunswick, Prince Edward Island, Newfoundland and Labrador, Northwest Territories, and Yukon (no documents) are all excluded from this table because these cases had no current policy documents that formally defined the concept of harm reduction

## Discussion

It is a critical time for harm reduction in Canada. As outlined earlier, federal political changes, a growing overdose epidemic, and increased advocacy efforts have opened a policy window with the potential to significantly advance evidence-informed harm reduction approaches, increase access to services, and promote the health and safety of people who use drugs across the provinces and territories. Despite this opportunity, how current provincial and territorial harm reduction policy frameworks (under which new policies and services may be developed and implemented) conceptualize and describe harm reduction has not been systematically analyzed. To address this gap, the present study assessed the quality of provincial and territorial policy frameworks, relative to the extent that they reflect internationally recognized harm reduction principles and attributes.

Our analysis reveals that harm reduction policy frameworks vary widely across jurisdictions in Canada and are conceptually weak overall. Two provinces, Quebec and British Columbia, account for 21 of the 54 current policy documents, and two jurisdictions, New Brunswick and Prince Edward Island, had only one current policy relevant to harm reduction. The Yukon Territory had none. We retrieved only two stand-alone provincial-level harm reduction policies [49, 50], indicating that policy commitments to harm reduction rarely occur outside broader discussions of other addiction/mental health/substance use or sexually transmitted and blood-borne infection issues in Canada. This finding is instructive in light of our observation that current harm reduction policy texts are dominated by rhetorical support for unspecified “harm reduction” services, in place of detailed discussion of any number of distinct interventions typically included under this approach.

Just as harm reduction services are often undifferentiated within provincial and territorial polices, core principles and attributes of harm reduction are generally weakly defined. Although many policies discuss harm reduction, only one document [49] met all 17 population and program indicators and reflected ideal harm reduction policy. Very few documents acknowledged that stigma and discrimination are issues faced by people who use drugs, that not all substance use is problematic, and that people who use drugs are legitimate participants in harm reduction policy development and implementation. Furthermore, despite widespread international acknowledgement of the need to specifically target programs to the needs of key populations [48], almost no documents discussed harm reduction for women, youth, Indigenous or LGBTQI people. Given this, it is unsurprising that the provinces and territories performed particularly poorly in specifying key harm reduction population characteristics. The highest-ranking province, British Columbia, scored just 42% overall.

With regard to program indicators, provinces fared somewhat better, with British Columbia again ranking highest with a score of 65%. However, we observed that most policy documents did not endorse many central program aspects of a harm reduction approach. A minority of documents recognized that abstaining from substance use is not required to receive services. Just over a quarter addressed the risk of drug overdose, and even fewer acknowledged the need to apply harm reduction approaches to an array of drugs and modes of use. The later omission is particularly striking in the context of several years of data suggesting increasing overdose deaths in Canada, including an overdose epidemic escalating dramatically in multiple provinces since 2013 [51–53].

A general dearth of in-depth discussion of what specifically constitutes a harm reduction approach to drug use may reflect the fact that harm reduction is considered by many to be a contested area of morality policy. Morality policies are domains of policymaking that are particularly resistant to instrumental-rational evidence because they often devolve into debates over deeply rooted values and political positions, rather than dispassionate consideration of data [54]. Harm reduction is considered a morality policy area because proponents and opponents construe harm reduction according to different normative beliefs about drug use (particularly the use of drugs that are illegal) and what constitutes the most appropriate approach for dealing with related negative health and social outcomes [54–56]. Canada has a long history of moral conflict over harm reduction and drug policy [57, 58]. Examples of past morality policy debates in the Canadian context include racial tensions precipitating the criminalization of opium in 1908 [59], opposition to implementation of Vancouver’s Insite [60, 61], enactment of municipal bylaws restricting methadone service provision [62], and forced closure of a fixed site syringe distribution program in Victoria, British Columbia [63].

In endorsing harm reduction, but failing to elaborate or affirm some of its most core (but perhaps most controversial) tenets—such as destigmatizing people who use drugs and involving them in policymaking—policymakers appear to be demonstrating reluctance to wade into a politically fraught arena. This may be especially true for the majority of policy documents (80%) reviewed here that were published during the 2006–2015 era, when prominent opponents of harm reduction held federal power. The implications of this hypothesis are of concern. Public policy documents, like other formal government texts, have communicative functions [64]. That is, they communicate to members of a given policy network—i.e., state and non-state actors who share an interest in specific areas of policymaking—in an attempt to alter behaviour of network members with divergent interests [64] and, in doing so, legitimize or illegitimatize responses to a given social concern. By endorsing harm reduction in name, but not in substance, provincial and territorial documents may be communicating a general lack of support for key aspects of the approach to a diverse array of policy stakeholders, and thereby indirectly to a broader public.

The present study has some key lessons for Canadian policymakers seeking to strengthen provincial and territorial harm reduction policy frameworks. First, further elaboration (and official endorsement) of key aspects of a harm reduction approach to illegal drug use is needed. This should include articulating clear formal definitions and developing stand-alone policies which discuss a range of harm reduction interventions in detail, and as distinct from other types of health and social services. One way to address this gap may be to leverage the common declaration that policies should be “evidence-based” or “evidence-informed” (endorsed by 70% of documents in our study) to incorporate additional research and expert perspectives into new documents. Second, there is a critical need for formal harm reduction policies to address overdose. A lack of policy in this area suggests that recent efforts to expand take-home naloxone programs may have been implemented ad hoc, rather than as part of comprehensive harm reduction policy frameworks aiming to effectively engage people who use drugs and reduce multiple drug-related harms. Third, it is important to note that though frameworks appear weak overall, Canadian policy documents do contain useful examples on which future policies can be modelled. We found that although rare, when provided, formal definitions of harm reduction were generally consistent with a number of the principles and attributes elaborated in our indicators. Additionally, British Columbia’s stand-alone *BC Harm Reduction Strategies and Services Policy and Guidelines 2014* [49] met all indicators included in our study and could serve as a particularly useful template for policymakers in other jurisdictions. This document also demonstrates that endorsing internationally recognized aspects of a harm reduction approach to illegal drugs is potentially politically feasible in the Canadian morality policy context.

The results of our study are generally consistent with previous CHARPP research, which demonstrated that historically, instrumental aspects of Canadian harm reduction policy frameworks are weak and do not consistently elaborate strong funding commitments, roles and responsibilities, timelines, or other governance structures [41]. As found here, our previous research noted that large parts of the Atlantic and Northern Canada had particularly poor policies overall. Taken together, these policy analyses suggest that Canada’s reputation as an early harm reduction pioneer is in part belied by weak policy commitments to harm reduction at the provincial and territorial level, where health services are mainly organized, administered, and funded.

### Strength and limitations

Although previous scholarship has documented federal harm reduction policy in Canada [8, 61, 65–68], almost no research attends to provincial and territorial harm reduction policy frameworks. Our study provides some of the first empirical data in this area. One of the strengths of our study includes our analysis of publicly available formal policy documents, which are designed to be aspirational and expressive, and convey government’s core messages and values in relation to a specific policy area. Their content can thus be expected to shape how both institutional actors and external stakeholders understand and communicate about policy issues. Further strengths include our comprehensive document retrieval procedures and involvement of stakeholders from across the country in a structured process to verify included policy documents. In addition, our standardized policy report card and CHARPP coding framework facilitated systematic cross-case descriptions of harm reduction conceptualizations using a common set of indicators derived from international standards. These methodological advances lend themselves well to within-country analyses, as demonstrated here, and also international comparative studies designed to describe how formal policies characterize harm reduction across nations, and over time.

A number of study limitations should also be noted. Document retrieval procedures focused on publicly available policy texts were obtained via a commercial search engine (Google). Not all formal provincial/territorial harm reduction policy documents may be available using this approach. Furthermore, our use of a public search engine precluded access to other types of internal government policy documents that could contain more detailed elaboration of harm reduction approaches, program features, and population characteristics. It is possible that more targeted strategies could recover additional documents (for example, requests made under freedom of information legislation, or analysis of provincial and territorial parliamentary records), and future research should explore whether other document retrieval methods augment the corpus of texts.

The current study focused on policy authored by provincial or territorial governments and delegated health authorities. While these bodies play a critical role in supporting the implementation of harm reduction in Canada, it should be emphasized that municipal, non-governmental, and federal bodies are often also influential. For example, in Ontario, many harm reduction services are funded by the provincial government but organized and delivered by municipal-level public health units [69]. Of note, the *BC Harm Reduction Strategies and Services Policy and Guidelines 2014* [49] was informed by a best practice document published in 2006 by a non-governmental harm reduction committee in Toronto, Ontario [70]. Additionally, recent formal policy changes at the federal level have eased the process for non-governmental, municipal, health authority, and provincial actors to secure federal exemptions to establish supervised consumption services [71]. This policy shift may influence future provincial and territorial policymaking related to this specific intervention. Both of these examples suggest a need for further research examining the role of municipal, non-governmental, and federal actors in harm reduction policymaking in Canada, including efforts to characterize how these policy levels interact and intersect with provincial and territorial authorities.

We assessed the quality of harm reduction policy documents and frameworks relative to the strength of their communicative functions. Other types of approaches are also valid for assessing policy quality. Indeed, we previously evaluated provincial and territorial harm reduction policies according to their governance functions [41]. Integration of population, program, and governance indicators into one analytic framework remains an outstanding task that would facilitate a more complete assessment of the quality of harm reduction policy frameworks in Canada.

Our focus on formal harm reduction policy frameworks enabled systematic analysis of aspects of harm reduction that are considered by policymakers to be politically and publicly feasible in Canada’s provinces and territories. However, Canada’s experience with harm reduction policymaking suggests that innovations in harm reduction service provision can occur in the absence of an explicit, supportive policy framework. For example, none of the current policy documents we analyzed from Alberta mentioned naloxone. Despite this, take-home naloxone kits have been available in Edmonton, Alberta’s capital city, since 2005 [6], and the provincial government funded and significantly expanded the program to additional cities in 2015 [22]. These observations demonstrate how analyses of formal policy documents provide only a partial, albeit important, perspective on the state of harm reduction in a given jurisdiction. In an attempt to address this gap, we are conducting additional research with key informants from across Canada’s provinces and territories to better understand the current scale and scope of harm reduction services, variations in political acceptance, and complexities of harm reduction policymaking in each jurisdiction.

## Conclusions

Despite the above limitations, the present study provided a systematic description of official language used to describe a harm reduction approach to illegal drugs across Canada. Our results documented both consistency and variability in provincial/territorial harm reduction policy frameworks and suggest that relatively few offer robust characterizations of harm reduction or go beyond rhetorical or generic support. Our approach is well suited to provide a high-level descriptive overview of how policymakers in different jurisdictions make sense of harm reduction. We conclude that despite increasing evidence of effectiveness of a harm reduction approach to problematic substance use, the quality of current policy in Canada is poor at the provincial and territorial level and does not specify key population or program features that are consistent with international understandings of the approach.

## Additional file

Additional file 1: Provincial and territorial policy documents and indicator scores; table contains all CHARPP indicator scores from 13 provincial and territorial harm reduction policy report cards. (XLSX 57 kb)

## Abbreviations

CHARPP: Canadian Harm Reduction Policy Project
LGBTQI: Lesbian, gay, bisexual, trans, queer and questioning, and intersex
